# Peri‐Implant Soft Tissue Deformities in the Anterior Maxilla: A Cross‐Sectional Study

**DOI:** 10.1111/cid.70168

**Published:** 2026-06-22

**Authors:** Emilio Couso‐Queiruga, Matteo Leoncini, Sophia E. Vollath, Gustavo Avila‐Ortiz, Ignacio Sanz‐Martin, Vivianne Chappuis, Manrique Fonseca, Clemens Raabe

**Affiliations:** ^1^ Department of Oral Surgery and Stomatology University of Bern School of Dental Medicine Bern Switzerland; ^2^ Department of Periodontics and Oral Medicine University of Michigan School of Dentistry Ann Arbor Michigan USA; ^3^ Private Practice Lausanne Switzerland; ^4^ Department of Reconstructive Dentistry and Gerodontology University of Bern School of Dental Medicine Bern Switzerland

**Keywords:** dental implants, esthetics, outcome assessment, outcome measures, phenotype

## Abstract

**Background:**

This study aimed to evaluate the prevalence of peri‐implant soft tissue deformities, including dehiscences (PSTDs) and deficiencies, around bone‐level (BL) and tissue‐level (TL) implants in the anterior maxilla, assess associated variables, and patient‐reported outcomes (PROs).

**Methods:**

Adults with implant‐supported prostheses (ISPs) in the anterior maxilla were recruited. Clinical and digital assessments were performed, and related variables were analyzed.

**Results:**

A total of 205 ISPs in 193 patients were evaluated. Mean follow‐up after implant placement was 11.3 ± 1.4 years. PSTD prevalence was comparable between BL and TL implants (88.8% vs. 94.4%; *p* = 0.19), or when PSTD ≥ 1 mm (23.9% vs. 22.5%; *p* = 0.97). Similarly, 99.25% and 100% of BL and TL showed deformities, *p* = 1.00. Prevalence of prosthetic interface/abutment (12.7% vs. 2.5%) and implant shoulder exposure (4.2% vs. 0%), and overcontoured ISP (35.2% vs. 12.7%) was significantly higher in TL implants (*p* < 0.01). Greater papilla dimensions and lower volume deficiency (90.3% vs. 98.6%; *p* < 0.01) were observed around BL implants, while mucosal discoloration was similar between groups (16.4% vs. 15.5%; *p* > 0.05). Greater PSTD depth was associated with older age, reduced keratinized mucosa width (< 2 mm), wider implant diameter, prosthetic overcontouring, lower mucosal volume, and papilla deficiencies (*p* < 0.05). Wider prosthetic emergence angles were associated with reduced papilla dimensions (*p* < 0.05). Thin mucosa (< 2 mm) was associated with grayish mucosal discoloration (OR = 2.79). Despite these findings, OHIP‐14 scores were low, and PROs were high, with no significant differences between groups.

**Conclusions:**

Prevalence of peri‐implant soft tissue deformities in the anterior maxilla is high irrespective of implant type and is associated with age, soft tissue phenotype, mucosal volume, papilla deficiencies, wider implant diameters, and prosthetic overcontouring. However, this does not seem to have a measurable impact on patient perception.

## 1 Introduction

Dental implant therapy has become a predictable and widely accepted treatment modality for replacing missing teeth, as reflected in its projected annual worldwide growth rate between 2025 and 2030 [1, 2]. When appropriate standards of case selection, multidisciplinary treatment planning, precise surgical and prosthetic execution, and individualized supportive peri‐implant care tailored to patient needs are followed, high long‐term survival rates can be achieved [3, 4, 5, 6, 7, 8, 9]. Nevertheless, even with adherence to these principles, biological complications such as peri‐implant diseases may compromise the success of implant therapy [10, 11, 12, 13]. Beyond inflammatory conditions such as peri‐implantitis, alterations of peri‐implant hard and soft tissues represent additional potential complications that may adversely affect clinical and esthetic outcomes of implant therapy [12, 14].

A deformity is a congenital or acquired alteration of the normal shape, size, or alignment of a biological structure [15]. Different components of the peri‐implant soft tissue phenotype [16] may be affected by proximal and non‐proximal deformities, including peri‐implant soft tissue dehiscences (PSTD) [17], also known as peri‐implant mucosal dehiscences [18] or peri‐implant marginal mucosa defects [19]. PSTDs have been defined as alterations of the peri‐implant soft tissue architecture characterized by an apical discrepancy of the mucosal margin with respect to its ideal position, with or without exposure of transmucosal prosthetic components or the implant fixture surface [19]. According to a recent systematic review and meta‐analysis, the estimated prevalence of PSTDs was 46.2%, with an incidence of up to 38.3% within the first 5 years following prosthetic loading [12].

Several risk variables for PSTDs have been identified, including implant malposition, immediate implant placement, inadequate prosthetic design, absence of or limited keratinized mucosa width (KMW), and reduced mucosal width (MT), no previous soft tissue augmentation, and marginal bone loss [12, 20, 21, 22, 23, 24, 25, 26, 27]. Nevertheless, evidence remains limited regarding the influence of specific implant designs, such as bone‐level (BL) versus tissue‐level (TL) implants, on the long‐term prevalence of PSTDs and other peri‐implant mucosal deformities [12]. Therefore, the primary aim of this cross‐sectional study was to evaluate the long‐term prevalence of PSTDs in the anterior maxilla between BL and TL titanium implants without a diagnosis of peri‐implantitis or implant malposition. Secondary aims were to identify potential factors associated with PSTD and other mucosal deformities, papilla height dimensions, and mucosal discoloration, as well as to evaluate patient‐reported outcomes (PROs).

## 2 Materials and Methods

### 2.1 Study Design, Ethical Approval, and Setting

This study was designed as a single‐center cross‐sectional clinical investigation that adhered to the Strengthening the Reporting of Observational Studies in Epidemiology (STROBE) guidelines [28]. The research protocol received approval from the ethical committee for clinical studies in the canton of Bern, Switzerland (KEK‐BE‐No. 2023‐01651), and was conducted in accordance with the Declaration of Helsinki as revised in 2013. Data were collected at the Department of Oral Surgery and Stomatology, School of Dental Medicine, University of Bern, Switzerland, between November 2023 and September 2024.

### 2.2 Recruitment

Adult participants with non‐molar, tooth‐bound, single implant‐supported prostheses (ISP) in need of a comprehensive dental evaluation were eligible to participate in this study [29]. Before inclusion, all potential participants were required to read, comprehend, and sign an informed consent form detailing the study's objectives and methodology. The inclusion criteria were: (1) ≥ 18 years old; (2) the presence of at least one BL or TL ISP in a non‐molar site in the anterior maxilla, placed in the Department of Oral Surgery and Stomatology at the University of Bern. The exclusion criteria were: (1) diagnosis or history of peri‐implantitis [30]; (2) implant malposition; (3) sites presenting anatomical conditions that precluded evaluation of the region of interest; and (4) any disabilities or barriers that may hinder comprehension, reading, or signing of the informed consent.

### 2.3 Clinical and Digital Acquisition

Intraoral photographs of the region of interest were obtained under consistent ambient lighting using a 100‐mm macro lens (Canon EF 100 mm f/2.8 L; Canon, Melville, NY, USA) mounted on a camera body (Canon EOS 80D) paired with a dual‐flash system (Speedlite 270EX II; Canon, Melville, NY, USA). Exposure settings were standardized at ISO 250, an aperture of f/25, and a shutter speed of 1/200. Additionally, an independent examiner captured a digital scan of the dental arch of interest using an intraoral scanner (Trios 5 Wireless; 3Shape, Copenhagen, Denmark), which was exported as a true‐color polygon (PLY) file.

All examinations conducted during the comprehensive oral evaluation were performed by two calibrated assessors (E.C.‐Q. and C.R.). The calibration process involved a discussion meeting to review the study protocol and a preliminary joint assessment of 10 randomly selected sites, ensuring consistency and standardization. Clinical measurements were made using a periodontal probe (Marquis probe; Hu‐Friedy, Chicago, USA), including probing depth (PD), bleeding on probing, and suppuration on probing at six sites around the dental implant and adjacent. Additionally, KMW was measured at the mid‐facial aspect as the distance from the mucosal margin to the mucosal junction in mm. Similarly, facial mucosal thickness (FMT) was directly measured utilizing a standard no. 20 endodontic finger spreader (Kerr, Kloten, Switzerland) at 3 mm apical to the mucosal margin, and perpendicular to the long axis of the ISP, without local anesthesia, as reported in previous studies [31]. Smoking status, history of periodontitis (yes/no), and frequency of supportive periodontal therapy (SPT) attendance were recorded.

Finally, a periapical radiograph centered on the region of interest was taken utilizing the parallel technique with film holders (Xios CG Supreme Size 2, Dentsply Sirona, Charlotte, USA). Patient demographic and dental history data, including age, sex, implant type (i.e., BL or TL implants), dimensions (i.e., diameter and length), platform, and type of ISP (cement‐ or screw‐retained), were also recorded.

### 2.4 Digital Imaging Analysis

PLY files were imported into software for further analysis (Geomagic Control X, 3D Systems, Rock Hill, SC, USA). Linear digital evaluations were conducted by two calibrated examiners (M.C. and S.E.V.), followed by a joint assessment of five randomly selected sites after a protocol review meeting with one of the coauthors (E.C.‐Q.). To ensure measurement consistency, both examiners independently assessed 10 sites of interest and repeated the measurements after a 1‐week interval, and if no agreement was observed among examiners, another investigator (E.C.‐Q) made the final decision.

**FIGURE 1:**
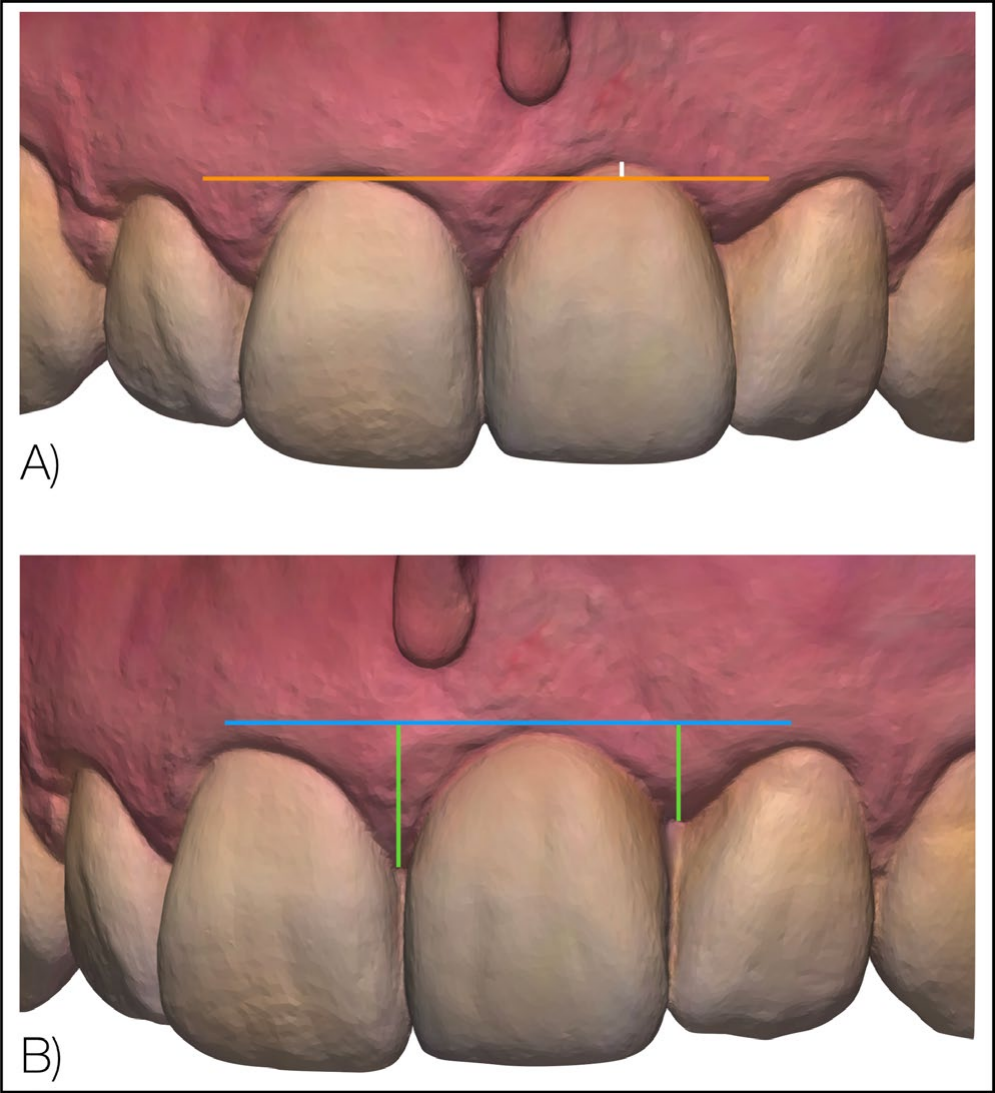
Schematic representation of the methodology used to assess peri‐implant soft‐tissue levels. (A) Evaluation of the mucosal margin position relative to the adjacent teeth. (B) Measurement of the distance from the mucosal margin to the most coronal point of the mesial and distal papilla.

For the assessment of PSTDs, a transversal line was drawn to determine the ideal position of the mucosal margin, considering the marginal gingiva of adjacent teeth for central incisors and premolars, and that on the contralateral homologous tooth for lateral incisors and/or canines. A PSTD was recorded as present when the mucosal margin was positioned apical to this reference line, whereas it was recorded as absent when the mucosal margin was at the same level as or coronal to this fiduciary mark. Subsequently, PSTD depth was recorded as the distance from this line to the mid‐facial mucosal margin position in mm. Similarly, the distance from the mid‐facial mucosal margin of the ISP to the most coronal tip of the mesial and distal papillae was also measured in mm. Figure 1 illustrates these methodological details.

**FIGURE 2:**
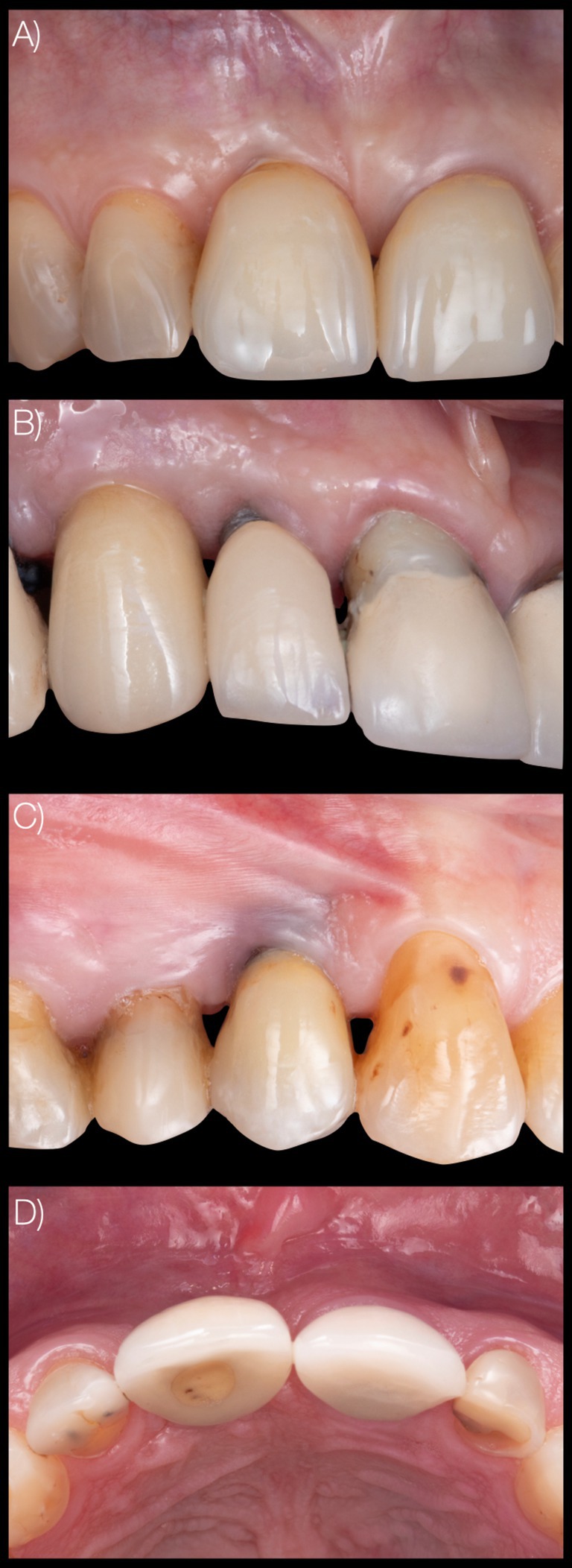
Examples of clinical situations demonstrating: (A) peri‐implant mucosal dehiscence with prosthetic abutment exposure; (B) peri‐implant mucosal dehiscence with implant shoulder exposure; (C) grayish mucosal discoloration; (D) occlusal view showing a soft‐tissue volume deficiency with a buccal concavity and an overcontoured implant‐supported prosthesis.

**FIGURE 3:**
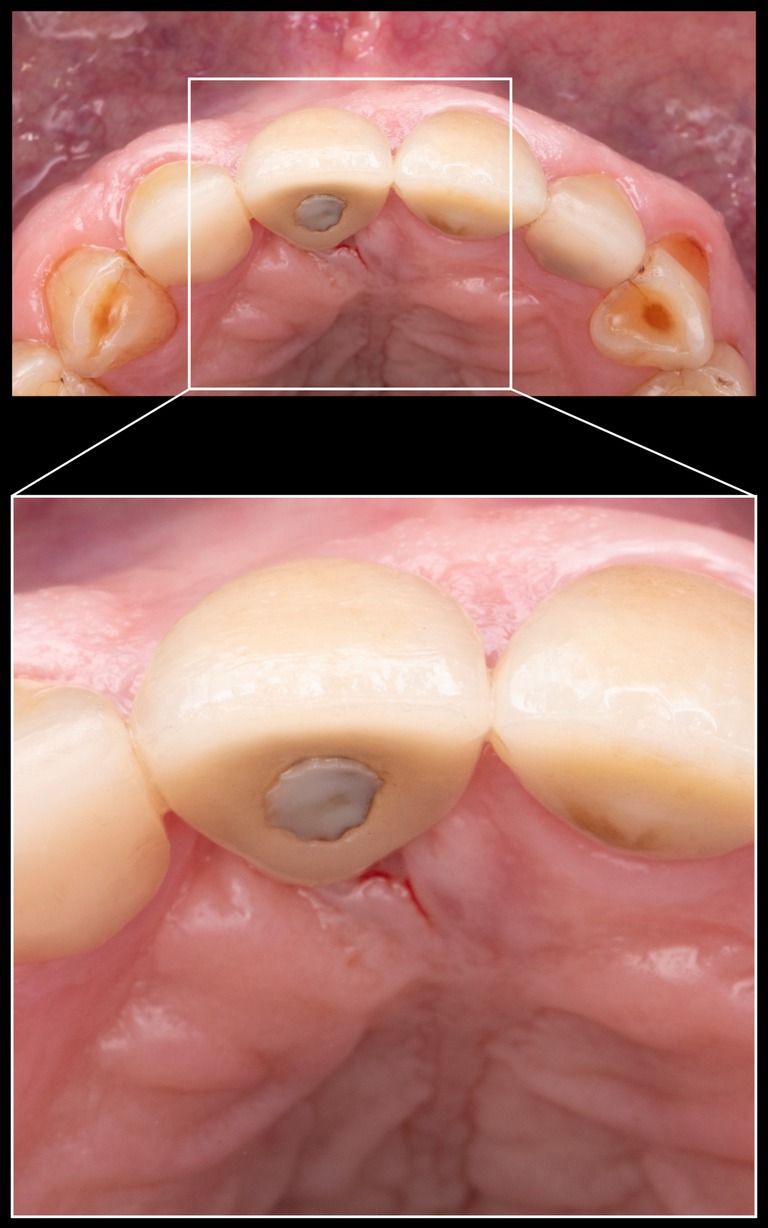
Representative example of a palatal mucosal cleft caused by traumatic flossing.

The presence of a soft tissue volume deficiency was assessed dichotomously (present/absent) at the most coronal part. A line connecting the buccal boundaries of the adjacent alveolar ridge at the mucosal margin and 1 mm apical to it, simulating the fully restored ridge contour, was used as the gold standard simulating adequate volume, and the distance from the most buccal part of the mid‐facial mucosa until this line was assessed in mm. Similarly, using the occlusal and frontal intraoral photographs, one examiner (M.C.) also evaluated the presence of mucosal cleft, Jemt papilla index [32], presence or absence of a grayish mucosal discoloration, or whether the ISP was overcontoured, as shown in Figures 2 and 3.

Finally, two independent evaluators (E.C.‐Q. and C.R.) imported the periapical radiographs into another software (ImageJ version 2, U.S. NIH, Bethesda, MD, USA) for the assessment of the mesial and distal prosthetic emergence geometry as reported elsewhere [33]. The calibration process was performed utilizing the first 10 consecutive patients until both inter‐ and intra‐examiner reliability were higher than or equal to 0.9. The angle and profile of the ISP emergence geometry were evaluated at two levels: level 1 (L1) corresponded to the implant level relative to the crestal bone (implant platform for BL implants, and the junction between the machined and microrough surface for TL implants). Level 2 (L2) corresponded to the prosthetic platform (abutment–crown interface for BL implants, and implant platform for TL implants) [33]. The height of the prosthetic platform was also recorded, corresponding to the abutment height in BL implants and the machined collar height in TL implants.

### 2.5 Predetermined Definitions

PSTD was defined, as previously described, as an apical migration of the mid‐facial peri‐implant mucosal margin of the ISP, assessed in relation to the ideal gingival margin of the adjacent tooth (for central incisors and premolars) or the contralateral tooth (for lateral incisors and/or canines), as reported elsewhere [12].

Peri‐implant soft tissue deficiency was defined as the presence of one or more deficiencies at ISPs, including a FMT < 2 mm, KMW < 2 mm, PSTD ≥ 1 mm, the presence of a buccal concavity, and/or papilla deficiency classified as Jemt class 0–II [32].

### 2.6 Patient‐Reported Outcome Measures

As reported elsewhere [34], patients completed the OHIP‐14 and an additional questionnaire with 100‐point visual analog scales (VAS) evaluating: (1) perceived similarity of the ISP to natural teeth; (2) functional ability; (3) esthetic satisfaction; (4) fulfillment of treatment expectations; (5) retrospective treatment tolerability perception (pain and discomfort); and (6) ability to perform oral hygiene. Questionnaires were completed independently by participants without examiner involvement to minimize potential Hawthorne effect bias.

### 2.7 Statistical Analysis

All analyses were performed using R statistical software (Version 4.3.2; R Foundation for Statistical Computing, Vienna, Austria), with the packages “tidyverse,” “robustlmm,” “glmmTMB,” “geepack,” “DHARMa,” “gtsummary,” and “DescTools.”

Patient‐ and implant‐related characteristics, as well as outcome variables, were descriptively summarized using mean values and standard deviations for continuous variables, and frequencies and percentages for categorical variables. Clinical, radiographic, and soft tissue‐related outcomes were further stratified by implant levels and tested for group differences.

Because some patients had more than one implant and to identify baseline parameters predictive of PSTD, variables were first screened univariately using robust linear mixed‐effects regression models, and those with *p*‐values ≤ 0.10 were entered into a final multivariable model. Note that robust mixed models were employed, as standard mixed models deviated from normality and were sensitive to outliers.

Generalized estimating equation (GEE) logistic regression models were applied to test univariable associations between explanatory variables and binary outcomes, including peri‐implant health, presence of a mucosal cleft, prosthetic‐related conditions, buccal concavity, overcontoured ISP, and grayish mucosal discoloration. To analyze univariable associations with count or low‐integer outcomes such as the OHIP‐14 score, zero‐inflated Poisson mixed regression models were used. For all other univariable analyses, robust linear mixed regression models were employed, as standard mixed models deviated from normality and were sensitive to outliers.

In all regression models, the patient was included as a random effect or as an exchangeable repeated measurement. Model fit was assessed using the DHARMa package for continuous and integer outcomes, and the Le Cessie–Van Houwelingen–Copas–Hosmer test for GEE logistic regression models. The multivariable models were additionally checked for collinearity. All *p*‐values ≤ 0.05 were considered statistically significant. No a priori sample size calculation was performed, as this was an exploratory, cross‐sectional analysis.

## 3 Results

### 3.1 Study Population and Sample Characteristics

A total of 193 participants with 205 ISPs were included in the study. Twelve patients contributed more than one ISPs. The population was comprised of 93 females (48.2%) and 100 males (51.8%), with a mean age of 59.0 ± 16.0 years. Most participants were non‐smokers (*n* = 165, 85.5%), while 28 (14.5%) were current or former smokers. Forty‐eight patients had a history of periodontitis (23.3%). SPT attendance was 0–1 sessions/year in 125 patients (64.8%), and 2–4 sessions/year in 68 (35.2%). The mean follow‐up time after implant placement was 11.3 ± 1.4 years. None of the implant sites had previously undergone soft tissue augmentation.

Of the 205 implants, 134 were BL (65.4%), while 71 were TL (34.6%). Implant diameters were 3.3 mm (*n* = 97, 47.3%) or 4.1 mm (*n* = 108, 52.7%), with lengths ranging from 8 to 14 mm. One hundred and twenty‐two ISPs (59.5%) were located in the incisor region, 10 (4.9%) in canine sites, and 73 (35.6%) in the premolar region. Most ISPs were screw‐retained (*n* = 176, 85.9%), the rest were cement‐retained (14.1%).

Mean PD values were 4.3 ± 0.8 mm for BL, and 4.5 ± 0.8 mm for TL implants (*p* = 0.66). Peri‐implant health was observed in 28 BL (20.9%) and 12 TL (16.9%) implants (*p* = 0.62). Mean KMW was greater for TL implants compared to BL implants (4.8 ± 1.5 mm vs. 3.2 ± 1.3 mm; *p* < 0.001), whereas mean FMT did not differ between both groups (BL: 2.3 ± 0.7 mm vs. TL: 2.2 ± 0.6 mm; *p* = 0.29).

Significant differences between BL and TL implants were observed for prosthetic and morphologic parameters. Statistically significant differences were observed regarding mesial angles L1 (BL: 19° ± 12°, TL: 5° ± 7°; *p* < 0.0001) and L2 (BL: 15° ± 9°, TL: 22° ± 9°; *p* < 0.0001), distal angles L1 (BL: 25° ± 8°, TL: 5° ± 7°; *p* < 0.0001) and L2 (BL: 19° ± 9°, TL: 18 ± 8; *p* = 0.001), and abutment/collar height (BL: 1.5 ± 0.8 mm, TL: 1.9 ± 0.3 mm; *p* < 0.0001) Mesial L1 and distal L1 angles were wider at BL implants (19° ± 12° and 25° ± 8°) compared with TL implants (5° ± 7° and 5° ± 7°; *p* < 0.0001). Conversely, mesial L2 angles were narrower at BL implants (15° ± 9° vs. 22° ± 9°; *p* < 0.0001), while distal L2 angles were comparable (19° ± 9° vs. 18° ± 8°; *p* = 0.001). Abutments were taller on TL implants than on BL implants (1.9 ± 0.3 mm vs. 1.5 ± 0.8 mm; *p* < 0.0001).

### 3.2 Inter‐Examiner Reliability

The ICC for all parameters ranged from 0.93 to 0.98 (95% CI: 0.90 to 0.99), indicating excellent inter‐rater reliability and agreement between examiners.

### 3.3 Peri‐Implant Soft Tissue Deformities and Variables of Interest

PSTD was highly prevalent in both groups, affecting 119 sites (88.8%) in the BL group and 67 sites (94.4%) in the TL group (*p* = 0.19). Exposure of the prosthetic abutment/interface was observed on 3 BL and 9 TL implant sites (2.5% vs. 12.7%; *p* = 0.004). A mucosal cleft, located either buccally or palatally, was identified around 3 BL implants and 6 TL implants (2.5% vs. 8.5%; *p* = 0.04). The implant shoulder was not visible on the buccal aspect of any BL implant, while it was detected on 3 TL implants (0% vs. 4.2%; *p* = 0.04).

Mean PSTD depth did not differ significantly between groups (BL: −0.5 ± 0.8 mm, range: −1.9 to 4.3 mm vs. TL: −0.7 ± 1.0 mm, range: −0.1 to 3.9 mm; *p* = 0.16). The Jemt papilla index on the mesial was comparable between groups (BL: 1.6 ± 0.7 vs. TL: 1.5 ± 0.8; *p* = 0.49), whereas the mean distal value was higher at BL implant sites (BL: 1.4 ± 0.7 vs. TL: 1.0 ± 0.8; *p* = 0.03). Linear papilla height measurements confirmed significantly greater papilla dimensions at BL implants at both mesial (3.2 ± 1.2 mm vs. 1.4 ± 0.7 mm; *p* = 0.008) and distal sites (2.5 ± 1.2 mm vs. 1.0 ± 0.8 mm; *p* = 0.0001).

When PSTD values were dichotomized as ≥ 1 mm and < 1 mm, 32 out of 134 BL implants (23.9%) with a mean value of 1.5 ± 0.46 mm, and 16 out of 71 TL implants (22.5%) exhibited PSTD values ≥ 1 mm with a mean value of 1.9 ± 0.96 mm (*p* = 0.97).

A soft‐tissue volume deficiency was frequently encountered in both groups (BL: 90.3% vs. TL: 98.6%; *p* = 0.09). At the mucosal margin level, the mean buccal concavity was similar (BL: −0.8 ± 0.5 mm, range: −2.6 to 0.2 mm vs. TL: −1.0 ± 0.6 mm, range: −2.6 to 0.2 mm; *p* = 0.58). One millimeter apical to the mucosal margin, the buccal concavity was greater around TL implants (BL: −0.7 ± 0.4 mm, range: −2.6 to 0 mm vs. TL: −1.0 ± 0.7 mm, range: −4.4 to 0 mm; *p* = 0.04). Prosthetic overcontouring was also more commonly observed on TL implants (BL: 12.7% vs. TL: 35.2%; *p* < 0.0001). A grayish mucosal discoloration was observed with similar frequency in both groups (BL: 16.4% vs. TL:15.5%; *p* = 0.86). Table 1 summarizes comparisons between BL and TL implant sites.

**TABLE 1:** Peri‐implant soft tissue deficiencies between bone and tissue‐level implants.

	Bone‐level	Tissue‐level	*p*
Peri‐implant soft tissue deficiencies and related‐parameters			
Peri‐implant soft tissue dehiscence	119 (88.8%)	67 (94.4%)	0.19
Exposure of the prosthetic abutment/interface	3 (2.5%)	9 (12.7%)	0.004*
Presence of the implant shoulder	0 (0%)	3 (4.2%)	0.04*
Mucosal cleft	3 (2.5%)	6 (8.5%)	0.04*
Mean mid‐facial mucosal margin (mm)	−0.5 ± 0.8 (ranging from −1.9 to 4.3)	−0.7 ± 1.0 (ranging from −0.1 to 3.9)	0.16
Mesial Jemt papilla index	1.6 ± 0.7	1.5 ± 0.8	0.49
Distal Jemt papilla index	1.4 ± 0.7	1.0 ± 0.8	0.03*
Mesial linear papilla height (mm)	3.2 ± 1.2 (ranging from 0.5 to 7)	1.4 ± 0.7 (ranging from −1.6 to 3.9 mm)	0.008*
Distal linear papilla height (mm)	2.5 ± 1.1 (ranging from 0 to 5.5)	1.0 ± 0.8 (ranging from 0 to 5.1)	0.0001*
Buccal concavity	121 (90.3%)	70 (98.6%)	0.09
Linear ridge deficiency at the mucosal margin (mm)	−0.8 ± 0.5 (ranging from −2.6 to 0.2)	−1.0 ± 0.6 (ranging from −2.7 to 0.2)	0.58
Linear ridge deficiency 1 mm apical to the mucosal margin (mm)	−0.7 ± 0.4 (ranging from −2.6 to 0)	−1.0 ± 0.7 (ranging from −4.4 to 0)	0.04*
Overcontoured prosthesis	17 (12.7%)	25 (35.2%)	< 0.0001*
Grayish mucosal discoloration	22 (16.4%)	11 (15.5%)	0.86

Abbreviation: mm, millimeters.

* *p* < 0.05.

Finally, regarding the composite outcomes related to peri‐implant soft tissue deformities, these were observed in 99.25% and 100% of BL and TL cases, respectively (*p* = 1.00).

### 3.4 Factors Associated With Peri‐Implant Soft Tissue Dehiscences

Univariable screening identified gender, age, implant diameter, and KMW as variables associated with the presence of PSTD (*p* ≤ 0.10) and, therefore, were eligible for further multivariable analyses.

For male patients, the predicted PSTD value was −0.14 mm, although this effect was not statistically significant (*p* = 0.23). Increasing age was significantly associated with apical migration of the mucosal margin, with an additional −0.01 mm per year (*p* = 0.02). In contrast, greater KMW was associated with a more coronal mucosal margin position, with a 0.09 mm coronal shift per additional millimeter of KMW (*p* = 0.009). Implant diameter showed a strong and significant effect: implants with a 4.1 mm diameter were associated with greater mean depth (−0.37 mm) compared with 3.3 mm implants (*p* = 0.002), as shown in Table 2.

**TABLE 2:** Multivariable analysis regarding factors affecting the mucosal margin level.

Mucosal margin level	Effect	*p*
Intercept	−0.29 (0.09, 0.48)	
Gender		
Female	Baseline	0.23
Male	−0.14 (−0.09, 0.38)	
Age	−0.01 (0.00, 0.02)	0.02*
Diameter		
3.3 mm	Baseline	0.002*
4.1 mm	−0.37 (0.14, 0.61)	
Keratinized mucosal width (4 mm)	0.09 (−0.17, −0.02)	0.009*

Abbreviation: mm, millimeters.

* *p* < 0.05.

Univariable analyses of variables of interest demonstrated significant associations between the mid‐facial mucosal margin and papilla dimensions. A one‐point decrease in mesial and distal Jemt papilla scores corresponded to an additional −0.17 mm (*p* = 0.03) and −0.26 mm (*p* < 0.0001) increases in PSTD depth on the mesial and distal, respectively. Presence of a buccal concavity was also associated with greater PSTD depth: each additional millimeter of concavity resulted in −0.42 mm (*p* = 0.0004) and −0.25 mm (*p* = 0.02) at the mucosal margin and at 1 mm apical to it, respectively.

Mesial papilla height was significantly associated with the mucosal margin position (*p* = 0.003), with an increase in PSTD depth of 0.13 mm per millimeter decrease in papilla height, whereas the distal papilla height showed no significant association (*p* = 0.27). Sites presenting overcontoured ISPs exhibited a significantly deeper mid‐facial PSTD (−0.55 mm, *p* = 0.0001). The result of the analysis of factors influencing the mid‐facial marginal mucosal level is summarized in Table 3.

**TABLE 3:** Univariable associations of factors influencing the mid‐facial marginal mucosal level.

Parameter	Effect on mucosal margin	*p*
Mucosal cleft	No: Baseline Yes: 0.36 (−0.16, 0.88)	0.17
Mesial papilla (Jemt classification)	−0.17 (−0.32, −0.01)	0.03*
Distal papilla (Jemt classification)	−0.26 (−0.40, −0.11)	< 0.0001*
Exposure of the prosthetic abutment/interface	No: Baseline Yes: 0.41 (−0.24, 0.38)	0.11
Grayish mucosal discoloration	No: Baseline Yes: 0.07 (−0.24, 0.38)	0.66
Mesial papilla height	0.13 (0.04, 0.22)	0.003*
Distal papilla height	0.05 (−0.04, 0.14)	0.27
Buccal concavity	No: Baseline Yes: 0.18 (−0.26, 0.62)	0.43
Buccal concavity 0 mm apical	0.42 (0.20, 0.64)	0.0004*
Buccal concavity 1 mm apical	0.25 (0.03, 0.46)	0.02*
Overcontoured prosthesis	No: Baseline Yes: 0.55 (0.27, 0.82)	0.0001*
Abutment height	0.08 (−0.06, 0.22)	0.26

* *p* < 0.05.

### 3.5 Factors Associated With Mesial and Distal Papilla Dimensions

For both mesial and distal papilla dimensions, prosthetic angles L1 and L2 demonstrated significant associations. Angle L1 showed a positive correlation with the mesial and distal papilla height (*p* = 0.002 and *p* < 0.0001, respectively), indicating a strong tendency toward taller papilla with increasing L1 values. In contrast, angle L2 was negatively associated with the distal papilla height (*p* = 0.005), while no significant association was observed for the mesial papilla (*p* = 0.18). Abutment height showed no significant relationship with either the mesial or distal papilla (*p* = 0.71 and *p* = 0.58, respectively), as shown in Table 4.

**TABLE 4:** Univariable associations of factors influencing the mesial and distal papilla level.

Parameter	Effect on mesial papilla distance	*p*	Effect on distal papilla distance	*p*
Abutment height	0.05 (−0.20, 0.29)	0.71	−0.07 (−0.30, 0.17)	0.58
Angle L1	0.03 (0.01, 0.04)	0.002*	0.03 (0.02, 0.05)	0.0001*
Angle L2	−0.02 (−0.04, 0.01)	0.18	−0.03 (−0.06, −0.01)	0.005*
Exposure of prosthetic abutment/interface	0.48 (−0.43, 1.38)	0.30	0.41 (−0.47, 1.28)	0.36
Buccal concavity	−0.55 (−1.32, 0.23)	0.17	−0.24 (−0.99, 0.51)	0.53
Buccal concavity 0 mm apical	0.08 (−0.33, 0.49)	0.71	0.02 (−0.37, 0.42)	0.90
Buccal concavity 1 mm apical	−0.32 (−0.70, 0.06)	0.10	−0.30 (−0.67, 0.06)	0.10
Overcontoured prosthesis	−0.06 (−0.56, 0.44)	0.82	−0.27 (−0.76, 0.21)	0.27

* *p* < 0.05.

### 3.6 Association Between Facial Mucosal Thickness and Grayish Mucosal Discoloration

Thin facial mucosa was significantly associated with the presence of grayish mucosal discoloration. Sites with a mucosa < 2 mm were associated with an odds ratio of 2.79 compared with sites presenting a thick mucosa (≥ 2 mm), indicating a 179% higher likelihood of discoloration in the thin phenotype group. In contrast, KMW did not reach statistical significance (*p* = 0.07), although a trend was observed: each additional millimeter of KMW was associated with a 25% reduction in the odds of grayish discoloration (OR = 0.75).

### 3.7 Patient‐Reported Outcomes

The mean OHIP‐14 scores were low and comparable between groups (1.30 ± 1.94 and 1.41 ± 2.1 for the BL and TL implants, respectively; *p* = 0.91). Patient‐reported satisfaction with the ISP was similarly high, with mean values of 92 ± 12 for BL and TL implants (*p* = 0.99).

Functional ability was rated slightly higher for BL implants (92 ± 13) than TL implants (86 ± 24), although the difference was not statistically significant (*p* = 0.81). Esthetic appearance received high ratings in both groups, with mean scores of 93 ± 11 and 90 ± 17 for BL and TL implants, respectively (*p* = 0.85).

Treatment expectations scores were met to a similar extent for BL (87 ± 16) and TL implants (87 ± 14; *p* = 0.96). Likewise, overall treatment tolerability in terms of pain and discomfort was comparable, with mean scores of 84 ± 20 and 84 ± 18 for BL and TL implants, respectively (*p* = 0.99). The ability to maintain adequate oral hygiene was rated favorably in both groups, with mean values of 94 ± 8 for BL and 93 ± 9 for TL implants (*p* = 0.86).

Finally, univariable analyses showed that the presence of PSTD or other soft tissue deformities, such as deficient interproximal papillary fill, has no significant effect on OHIP‐14 and VAS scores (*p* > 0.38).

## 4 Discussion

This cross‐sectional study provides a comprehensive analysis of peri‐implant soft tissue deformities associated with BL and TL implants in the anterior maxillary region, including identification of potential factors associated with PSTDs, papilla height dimensions, and grayish mucosal discoloration, as well as evaluation of the effect of PSTD on PROs. After a mean follow‐up exceeding 10 years, PSTDs were observed to be highly prevalent in the anterior esthetic zone.

Although PSTD depth did not differ significantly between implant types, TL implants more frequently exhibited mucosal clefts, prosthetic interface exposure, and implant shoulder exposure. These observations could be attributed to the considerably greater soft‐tissue deficiency, together with the increased overcontouring of ISPs observed in the TL group, suggesting a correlation between implant design and prosthetic morphology. To the best of our knowledge, this is the first study to compare BL and TL digital imaging outcomes in the anterior esthetic zone after a prolonged functional period. The prevalence of PSTDs observed in the present study is higher than the 54.2% reported by Tavelli et al. in a cross‐sectional study with a shorter follow‐up. In contrast, the prevalence of abutment or implant exposure in our cohort was considerably lower than the 74% reported in their study [25]. Nonetheless, our findings are consistent with a recent systematic review and meta‐analysis indicating that both the incidence and depth of PSTD increase over time [12], and also with the outcomes reported in other cross‐sectional studies [25, 35, 36, 37, 38]. The observed discrepancies among studies may be attributed to differences in study methodology, eligibility criteria, 3D implant position, intraoral location, and follow‐up period.

To the best of our knowledge, no other long‐term studies have compared papillary dimensions between BL and TL implants in the anterior esthetic zone. Significantly reduced papillary dimensions were observed around TL implants and at distal sites, independently of implant type. This finding may be related to the increased overcontouring of ISPs, the limited possibility to modify the prosthetic emergence profile when required, reducing prosthetic management flexibility, and the supracrestal position of the implant–abutment interface. Interestingly, the pattern observed at distal papillae has been previously reported in a commentary article related to the central incisor area [39] and has been attributed to post‐extraction dimensional changes influenced by the treatment modality and management of the extraction site, as well as anatomical and phenotypical characteristics [40, 41, 42, 43, 44], other site‐specific anatomical conditions [16, 45, 46], and surgical and prosthetic variables [21, 47, 48, 49, 50, 51, 52]. These papillae dimensions could be improved through soft tissue augmentation procedures, depending on the specific clinical situation [53]. Interestingly, in the present study, prosthetic emergence angles showed a significant correlation with mesial and distal papillary height, with wider emergence angles associated with reduced papillary dimensions. While previous studies have linked wider emergence angles to a higher risk of peri‐implant diseases [6, 7, 54], to the best of our knowledge, the present study is the first to demonstrate an association between emergence angle and papilla height. These findings underscore the importance of appropriate prosthetic design and accurate apico‐coronal implant positioning, particularly in the anterior esthetic zone.

Risk indicators for the presence of PSTD included older age, reduced KMW, and wider implant diameter. Similarly, both lower mesial and distal Jemt papilla scores, increased buccal concavity, reduced papilla height, and overcontoured ISPs showed significant positive associations with more apical positioning of the mid‐facial mucosal margin. Previous studies have also reported associations between reduced KMW and the presence and severity of PSTD [25, 55, 56, 57], as well as with a thin peri‐implant soft‐tissue phenotype [22, 24, 58, 59], longer time in function [25], lower papilla esthetic index scores [22], soft tissue volume deficiency [14, 60], and a buccally convex emergence profile [61, 62]. However, not all prosthetic design variables seem to have a consistent influence. A recent randomized controlled trial did not detect significant differences between differences divergent or linear abutment morphologies [63]. The higher prevalence of PSTD associated with wider implant diameters, together with the reported higher prevalence of peri‐implantitis [29], emphasizes the importance of selecting the narrowest possible implant diameter compatible with functional and esthetic requirements in the anterior zone [64]. Notably, implants with a thin facial mucosa (< 2 mm) demonstrated a 179% higher likelihood of exhibiting grayish mucosal discoloration. These findings align with prior evidence demonstrating that abutment shade does not significantly influence peri‐implant mucosal color in sites exhibiting a mucosal thickness of ≥ 2 mm [65, 66, 67]. This evidence explains why alternative treatment strategies have been proposed and explored for sites with a thin peri‐implant mucosal phenotype [68, 69, 70, 71].

Despite the high prevalence of peri‐implant soft‐tissue deformities observed in this study, PROs assessed with OHIP‐14 scores and VAS were generally associated with patient satisfaction. This may explain why some studies have reported minimal changes in patient‐perceived esthetic satisfaction following the treatment of PSTD in both the short and long term [72, 73]. Nevertheless, a recent systematic review suggested that peri‐implant soft tissue augmentation may positively influence both professional‐ and patient‐reported esthetic outcomes [74]. These findings highlight the limited impact of PSTD in the anterior esthetic zone from the patient's perspective. Therefore, unless treatment is required by the patient, clinically indicated, or PSTD is expected to influence the onset or progression of other deformities or peri‐implant diseases, therapeutic interventions should be tailored to the specific clinical situation and guided by a patient‐centered approach [18].

Despite adherence to high methodological standards, this study has several limitations. First, the sample was restricted to non‐molar ISPs with no clinical or radiographic signs of peri‐implantitis and with a generally adequate three‐dimensional implant position. Therefore, excluding cases of implant malposition and peri‐implantitis, which are frequently associated with PSTD and other deficiencies. While this strict selection enhances internal validity, it may limit the generalizability of the findings to other reconstruction types, anatomical locations, or sites affected by peri‐implantitis. Second, only BL and TL implants from a single system were included. This approach allowed for a more controlled comparison but may limit the applicability of the results to other implant systems. Third, a higher number of BL implants (*n* = 134) compared with TL implants (*n* = 71), and with no prior sample size calculation, were included. This imbalance reflects the trends in clinical protocols in the study center, where BL implants are more frequently placed in the anterior maxillary region. Nevertheless, a substantial sample of TL implants placed in the same anatomical region was analyzed, which strengthens the relevance of the findings for this specific implant type. This could also explain the differences in KMW between BL and TL implants, and the potential selection bias. Fourth, the strict criteria applied to determine the presence of PSTD and its digital analysis may have overestimated its prevalence. However, when dichotomized at ≥ 1 mm, approximately 25% of the implants still demonstrated PSTD values equal to or greater than this threshold. Fifth, the method used to assess FMT may be subject to measurement inaccuracy and limited reproducibility. In addition, although no local anesthesia was administered, the procedure may have caused some degree of patient discomfort. Finally, the cross‐sectional design and sample size exclude causal inference and may have limited the ability to detect certain associations. Future studies on this topic should be conducted using standardized and reliable assessment methods, larger and more balanced samples, multiple implant systems, a broader range of clinical scenarios (i.e., soft tissue augmentation procedures), anatomical locations, and 3D implant positioning. Similarly, robust multivariable analyses should be employed to further elucidate the complex interrelationships and potential collinearity among different clinical and radiographic parameters.

## 5 Conclusions

Within the limitations of this cross‐sectional study, the following conclusions can be drawn:

– PSTDs were highly prevalent in the maxillary anterior region more than 10 years after loading.
– TL implants were associated with a higher prevalence of PSTDs and depth, greater exposure of the prosthetic interface and implant shoulder, lower papilla dimensions, more pronounced buccal volume deficiency at the most coronal region, and a higher frequency of overcontoured ISPs.
– Greater PSTD depth was associated with older age, reduced keratinized mucosa, wider implant diameter, overcontoured ISPs, and the presence of volume and papilla deficiencies.
– Wider prosthetic emergence angles were associated with reduced papilla dimensions.
– Thin facial mucosa (< 2 mm) was significantly associated with grayish mucosal discoloration.
– Despite the high prevalence of PSTD and soft tissue deficiencies, PROs indicated high patient satisfaction with function, esthetics, treatment expectations, treatment tolerability, and oral hygiene maintenance.

## Author Contributions

E.C.‐Q. conceived and designed the project. E.C.‐Q., M.F., and C.R. contributed to data acquisition. E.C.‐Q., M.L., and S.E.V. participated in digital data analysis. E.C.‐Q. led the writing, while M.L., S.E.V., G.A.‐O., I.S.‐M., M.F., V.C., and C.R. critically revised the manuscript. All authors provided final approval and agreed to be accountable for all aspects of the scientific work.

## Funding

This research project was partially funded by a grant from the International Team for Implantology (ITI) awarded to Drs. Emilio Couso‐Queiruga and Clemens Raabe, project ID: 1905‐2024.

## Ethics Statement

The study protocol received approval from the ethical committee for clinical studies in the canton of Bern, Switzerland (KEK‐BE‐No. 2023‐01651).

## Conflicts of Interest

The authors declare no conflicts of interest.

## Data Availability

The data that support the findings of this study are available on request from the corresponding author. The data are not publicly available due to privacy or ethical restrictions.
